# Myelin-reactive antibodies initiate T cell-mediated CNS autoimmune disease by opsonization of endogenous antigen

**DOI:** 10.1007/s00401-016-1559-8

**Published:** 2016-03-29

**Authors:** Silke Kinzel, Klaus Lehmann-Horn, Sebastian Torke, Darius Häusler, Anne Winkler, Christine Stadelmann, Natalie Payne, Linda Feldmann, Albert Saiz, Markus Reindl, Patrice H. Lalive, Claude C. Bernard, Wolfgang Brück, Martin S. Weber

**Affiliations:** Department of Neuropathology, University Medical Center, Georg August University, Göttingen, Germany; Department of Neurology, Klinikum rechts der Isar, Technische Universität München and Munich Cluster for Systems Neurology, Munich, Germany; Monash Regenerative Medicine Institute, Multiple Sclerosis Research Group, Monash University, Melbourne, Australia; Service of Neurology, Hospital Clinic, University of Barcelona, Barcelona, Spain; Clinical Department of Neurology, Medical University of Innsbruck, Innsbruck, Austria; Division of Neurology, Department of Clinical Neurosciences, University Hospital of Geneva, Geneva, Switzerland; Department of Pathology and Immunology, Faculty of Medicine, University Hospital of Geneva, Geneva, Switzerland; Department of Neurology, University Medical Center, Georg August University, Göttingen, Germany

**Keywords:** Auto-antibodies, Opsonization, Myeloid antigen-presenting cells, Fc receptor, Experimental autoimmune encephalomyelitis, Multiple sclerosis

## Abstract

In the pathogenesis of central nervous system (CNS) demyelinating disorders, antigen-specific B cells are implicated to act as potent antigen-presenting cells (APC), eliciting waves of inflammatory CNS infiltration. Here, we provide the first evidence that CNS-reactive antibodies (Ab) are similarly capable of initiating an encephalitogenic immune response by targeting endogenous CNS antigen to otherwise inert myeloid APC. In a transgenic mouse model, constitutive production of Ab against myelin oligodendrocyte glycoprotein (MOG) was sufficient to promote spontaneous experimental autoimmune encephalomyelitis (EAE) in the absence of B cells, when mice endogenously contained MOG-recognizing T cells. Adoptive transfer studies corroborated that anti-MOG Ab triggered activation and expansion of peripheral MOG-specific T cells in an Fc-dependent manner, subsequently causing EAE. To evaluate the underlying mechanism, anti-MOG Ab were added to a co-culture of myeloid APC and MOG-specific T cells. At otherwise undetected concentrations, anti-MOG Ab enabled Fc-mediated APC recognition of intact MOG; internalized, processed and presented MOG activated naïve T cells to differentiate in an encephalitogenic manner. In a series of translational experiments, anti-MOG Ab from two patients with an acute flare of CNS inflammation likewise facilitated detection of human MOG. Jointly, these observations highlight Ab-mediated opsonization of endogenous CNS auto-antigen as a novel disease- and/or relapse-triggering mechanism in CNS demyelinating disorders.

**Electronic supplementary material:**

The online version of this article (doi:10.1007/s00401-016-1559-8) contains supplementary material, which is available to authorized users.

## Introduction

B cells, plasma cells and antibodies (Ab) are increasingly recognized as key players in inflammatory central nervous system (CNS) demyelinating diseases, such as multiple sclerosis (MS), neuromyelitis optica (NMO) and related disorders. Within the cerebrospinal fluid of the majority of MS patients, locally supported plasma cells continuously produce oligoclonal immunoglobulin (Ig) [36, 51], which remain a hallmark diagnostic finding. B and plasma cells are commonly found in MS lesions [41] and Ab deposition co-localizes with complement activation and ongoing demyelination [16]. In NMO, compelling evidence suggests that anti-aquaporin (AQP)-4-Ab selectively target astrocytes resulting in subsequent demyelination [29].

B cells constitutively express major histocompatibility complex (MHC) class II and act as powerful antigen-presenting cells (APC) when they recognize conformational protein antigen via their B cell receptor (BCR) [9]. B cells from MS patients reveal signs of chronic activation with a differential shift toward antigen-experienced memory B cells producing pro-inflammatory cytokines, such as interleukin-6 [12] and granulocyte-macrophage colony-stimulating factor (GM-CSF) [25]. These properties, along with the fulminant success of the clinical trials testing anti-CD20 monoclonal Ab [18, 19], suggest that antigen-experienced B cells may act as potent APC in MS.

A series of recent experimental investigations aimed to directly address the role of B cells, plasma cells and Ab in development of inflammatory CNS demyelinating disease. First, transgenic mice were generated in which B cells recognize myelin oligodendrocyte protein (MOG) and plasma cells constitutively produce high titers of pathogenic anti-MOG Ab (Th mice); upon active immunization, these mice showed a fulminant course of experimental autoimmune encephalomyelitis (EAE) with enhanced CNS demyelination [27]. When Th mice were further crossed with MOG T cell receptor (TCR) transgenic mice (2D2 mice) [4], the resulting line (Thx2D2) even spontaneously developed EAE [3, 21]. A similar observation was reported on the SJL/J background, furthermore, demonstrating that transgenic T cells can recruit endogenous MOG-specific B cells [39]. In an attempt to elucidate which immunological components were required for spontaneous EAE development, a pivotal recent report demonstrated that myelin-recognizing B and T cells sufficed to trigger EAE development in C57BL/6 mice [34], corroborating that auto-reactive B cells are an essential APC population in this model.

Notwithstanding these results, we here report on a crucial complementary role of CNS-reactive Ab likely completing the scenario how initial recognition of auto-antigen in development of CNS autoimmune disease can occur. We show that traces of CNS antigen are opsonized by myelin-reactive Ab, making them recognizable for Fc receptor carrying myeloid APC. Subsequent to internalization, processed myelin antigen is presented to myelin-recognizing T cells triggering their expansion and encephalitogenic differentiation. We demonstrate that this mechanism indeed sparks experimental CNS autoimmunity; first, we show that in the absence of B cells, Thx2D2 mice develop spontaneous EAE indistinguishable from its course in mice containing B cells. Second, and most importantly, adoptive transfer of serum from Th mice or of purified anti-MOG Ab 8.18C5 into naïve 2D2 recipients triggered activation and expansion of T cells followed by severe and robust EAE, suggesting that the mechanism of Ab-mediated opsonization of auto-antigen may indeed contribute to initiation and propagation of CNS demyelinating disease.

## Materials and methods

### Mice

MOG p35-55 TCR transgenic 2D2 mice were kindly provided by Dr. Kuchroo (Boston, USA). MOG Ig heavy chain knockin (Th) mice were kindly provided by Dr. Wekerle (Munich, Germany). Fcγ receptor knockout (FcγR^−/−^) mice were kindly provided by Dr. Nimmerjahn (Erlangen, Germany). Wild-type (WT) C57BL/6 mice were purchased from Charles River (Sulzfeld, Germany). All murine experiments were carried out as approved by the government of Upper Bavaria (protocol number 55.2-1-54-2531-67-09) and the government of lower Saxony (protocol number 33.9-42502).

### B cell depletion

B cell depletion was achieved by weekly intraperitoneal (i.p.) injections of 200 µg of murine anti-CD20 or anti-ragweed isotype control Ab (both provided by Genentech, South San Francisco, USA) in 200 µl PBS.

### Antigens and EAE induction regiments

Mouse MOG peptide (p) 35-55 (MEVGWYRSPFSRVVHLYRNGK) was synthesized by Auspep (Parkville, Australia). Murine MOG protein 1-117 (mMOG) and human MOG protein (hMOG) were kindly provided by C.C.A. Bernard and synthesized, purified and refolded as previously reported [8]. 8–10 week-old-female Th or WT mice were immunized subcutaneously with either a suboptimal dose of 35 µg mMOG or an optimal dose of 50 µg mMOG in Complete Freund’s Adjuvant (CFA) followed by 200 ng of pertussis toxin (Sigma-Aldrich, St. Louis, USA) i.p. at the day of immunization and 2 days thereafter. Alternatively, WT mice were immunized with 100 µg MOG p35-55 in CFA followed by two injections of 300 ng pertussis toxin. For spontaneous EAE experiments, Th mice were bred with 2D2 mice (Thx2D2). To induce EAE by transfer of MOG-specific Ab, a serum preparation containing anti-MOG Ab or monoclonal Ab clone 8.18C5 were injected into the tail vein of WT or 2D2 recipients. 150 µl Th serum or control serum was injected twice a week up to a total of five injections; 150 µg 8.18C5 Ab or isotype control Ab (clone: MOPC-21, Bio X cell, West Lebanon, USA) was injected twice a week up to a total of ten injections. In recipients, no immunization or adjuvant treatment was added.

### Evaluation of EAE

Mice were assessed for clinical signs of EAE as follows: 0 = no clinical disease, 1 = tail weakness, 2 = hind limb weakness, 3 = one paralyzed hind limb, 4 = two paralyzed hind limbs, 5 = moribund or dead. To evaluate balance and general motor function the elevated beam test was used; mice were placed on a raised beam with a maximal height of 40–50 cm and a length of 100 cm and the time needed to traverse was measured. Mice were evaluated daily starting 2 weeks prior to the respective treatment.

### Intrathecal injection of mMOG

Mice were injected with 40 µg mMOG in 10 µl PBS or 10 µl of 10 % Evans blue percutaneously into the cisterna magna with a 30-gauge needle in 45° anteflexion of the head.

### Generation of anti-MOG Ab containing serum

Serum containing high titers of pathogenic anti-MOG Ab or control serum was obtained from Th mice immunized with 100 µg mMOG (Th serum) or from WT mice immunized with 100 µg MOG p35-55 (control serum), respectively. Donor mice were immunized to ensure the maximum capacity of obtained myelin-specific Ab. 14 days after immunization, blood was obtained by puncture of the left ventricle; serum was separated by centrifugation, pooled for further use and stored at −20 °C.

### Preparation and digestion of 8.18C5

Anti-MOG monoclonal Ab clone 8.18C5 was generated by hybridoma cells kindly provided by Dr. Linington (Glasgow, UK). Hybridoma cells were cultured in complete medium (RPMI, 5–10 % fetal calf serum, 50 U/ml penicillin, 50 µg/ml streptomycin, 2 mM l-glutamine, 1 mM sodium pyruvate, 0.05 mM β-mercaptoethanol) in large-scale flasks (Greiner bio-one, Kremsmuenster, Austria) and Ab was purified using rProtein A/Protein G Sepharose columns (rProtein A/Protein G GraviTrap, GE Healthcare, Little Chalfont, UK), according to manufacturer's recommendations. 8.18C5 IgG was digested by ficin (Pierce Mouse IgG1 Fab and F(ab′)_2_ Preparation Kit, Thermo Scientific, Waltham, USA), according to manufacturer’s recommendations; integrity and binding capacity of 8.18C5 Ab and resulting 8.18C5 F(ab′)_2_ fragments were verified by 6 % sodium dodecyl sulfate polyacrylamide gel electrophoresis (SDS-PAGE) under non-reducing conditions, followed by protein staining with Coomassie brilliant blue G250 (Biorad, Munich, Germany) and competitive anti-MOG enzyme linked immunosorbent assay (ELISA), respectively.

### IgG preparation from patients with demyelinating CNS inflammation

IgG Ab preparations were isolated by Protein G Sepharose 4 Fast Flow (GE Healthcare) from plasma exchange fluid obtained from two patients with neuromyelitis optica spectrum disorders (NMOSD), and plasma from one healthy blood donor. One patient was a 62-year-old Caucasian woman with past history of relapsing bilateral optic neuritis. The patient was admitted because of a severe brainstem relapse that was treated with corticosteroids and plasma exchange followed by rituximab. The other patient was a 55-year-old Caucasian woman with a relapsing disease since the age of 48, who presented with a severe optic neuritis relapse in January 2016 that was treated with corticosteroids and plasma exchange followed by rituximab. In both cases, cerebrospinal fluid showed no oligoclonal bands, and serum samples were negative for anti-AQP-4 Ab but positive for anti-MOG Ab (titer 1:1280, patient #1; 1:5120, patient #2) measured by a recombinant live cell-based immunofluorescence assay with HEK293 cells as previously described [30]. Prior to our in vitro assay, the general ability to bind soluble recombinant hMOG was tested by ELISA using an anti-MOG Ab positive IgG preparation. The healthy blood donor was tested anti-MOG Ab negative, and IgG preparation served as negative control. Samples were kindly provided by Dr. Reindl (Innsbruck, Austria) and Dr. Saiz (Barcelona, Spain). The use of the patient’s material was approved by the Ethic Committee of the Hospital Clinic of Barcelona, written consent was obtained.

### Fc blocking in vivo

To block Fcγ receptors in vivo, mice were injected i.p. daily with 100 µg anti-CD16/CD32 Ab (Clone: 2.4G2, TONBO bioscience, San Diego, USA) in 100 µl 1xPBS starting 2 days prior to further treatment.

### Assessment of in vivo proliferation of T cells

In vivo proliferation of T cells was evaluated by i.p injection of 200 µl Bromdesoxyuridin (BrdU; 10 mg/ml) 24 h before flow cytometry evaluation using a BrdU Flow kit (BD Pharmingen, San Diego, USA), according to manufacturer´s recommendations.

### Generation of bone marrow-derived myeloid APC

To generate bone marrow-derived macrophages (BMDM), bone marrow isolated from hind limbs of WT or FcγR^−/−^ mice was cultured at 37 °C for 7 days in medium containing 30 % conditioned L929 cell supernatant (DMEM, 30 % L929 supernatant, 10 % fetal calf serum, 5 % horse serum, 50 U/ml penicillin, 50 µg/ml streptomycin, 0.05 mM β-mercaptoethanol). For bone marrow-derived dendritic cell (BMDC) generation, bone marrow was isolated from hind limbs of WT mice and cultured for 7 days in complete medium (RPMI, 10 % fetal calf serum, 50 U/ml penicillin, 50 µg/ml streptomycin, 2 mM l-glutamine, 1 mM sodium pyruvate, 0.05 mM β-mercaptoethanol) containing 25 ng/ml GM-CSF (Sigma-Aldrich).

### Evaluation of antigen uptake by myeloid APC

Adherent BMDM or BMDC were harvested using cell scraper, 0.5 × 10^5^ cells/well were plated into 96-well flat-bottom plates (Sarstedt, Nuembrecht, Germany) and pre-stimulated with 500 ng/ml lipopolysaccharide (LPS, Sigma-Aldrich). 24 h thereafter, cells were incubated for 2 h with DyLight-405 labeled conformational mMOG (mMOG-DyLight-405) or ovalbumin (OVA)-FITC (Life Technologies, Thermo Fisher Scientific) in the presence of 50 µg/ml 8.18C5 Ab, 8.18C5 F(ab′)_2_ fragment, anti-OVA Ab (clone: TOSG1C6, BioLegend, San Diego, USA) or isotype control Ab (clone: MOPC-21). To investigate phagocytosis of conformational hMOG, murine BMDM which are known to recognize human IgG via their Fc receptor [37] were incubated with hMOG-DyLight-405 for 2 h in the presence of 15 µg/ml of the respective IgG preparation. Where indicated, Fcγ receptors were blocked using anti-mouse CD16/CD32 Ab (Clone: 2.4G2, 1:100 dilution).

### Assessment of T cell proliferation and differentiation in vitro

Adherent BMDM were harvested using cell scraper, 0.5 × 10^5^ cells/well were plated into 96-well flat-bottom plates and pre-stimulated with 50 ng/ml LPS and mMOG in the presence of 50 µg/ml 8.18C5 Ab, 8.18C5 F(ab′)_2_ fragment or isotype control Ab. Where indicated, Fcγ receptors were blocked using anti-mouse CD16/CD32 Ab (Clone: 2.4G2, 1:100 dilution). 24 h thereafter, 1 × 10^5^ MACS-purified (Pan T cell Isolation Kit, Miltenyi, Bergisch Gladbach, Germany) carboxyfluorescein succinimidyl ester (CFSE)-stained (CFSE Cell Division Tracker Kit, BioLegend) or unstained T cells from 2D2 mice were added per well. 72 h thereafter, T cell proliferation and differentiation were evaluated by flow cytometry or ELISA, respectively.

### Flow cytometric analysis

To obtain single cell suspensions of lymphoid tissues, respective spleens and lymph nodes were carefully dissected and passed through 70 µm strainer. Freshly obtained blood was mixed 1:2 with 1 mM EDTA and erythrocytes were lysed using BD Pharm Lyse Buffer, according to manufacturer’s recommendation. B cells were stained for B220 (BioLegend) CD19 (BioLegend) and/or CD20 (BioLegend), T cells for CD3 and/or CD4 (all BD Bioscience). T cell activation was determined by expression of CD69 (BD Bioscience). T cell differentiation was evaluated by intracellular cytokine staining for IFN-γ (eBioscience, San Diego, USA) and IL-17A (BD Bioscience) after 4-h incubation with phorbol 12-myristate 13-acetate (PMA, 50 ng/ml) and ionomycin (0.5 μg/ml) in the presence of monensin (1 µl Golgi-Stop per ml cell suspension, BD Bioscience). Dead cells were excluded using an Aqua Dead Cell Stain Kit (Invitrogen, Thermo Fisher Scientific, 405 nm excitation). CNS-infiltrating cells were isolated by discontinuous density gradient (Percoll) [24] and stained in a similar manner for CD3, CD4, IFN-γ and IL-17A. Myeloid APC were stained for CD11b and CD11c (both BioLegend). Expression of Fcγ receptor (FcγR) I and III of BMDM and BMDC was determined using anti-CD64 Ab (FcγRI, BioLegend) and anti-CD16 Ab (FcγRIII, BioLegend). APC secretion of IL-1α and IL-23 (both BioLegend) was determined after 5-h incubation with 500 ng/ml LPS in the presence of monensin (1 µl Golgi-Stop per ml cell suspension. BD Bioscience).

### ELISA for cytokine analysis

ELISA for IFN-γ, IL-17 or GM-CSF was performed using paired monoclonal Ab per manufacturer’s recommendations (DuoSet, R&D Systems, Wiesbaden‐Nordstadt, Germany). The results are expressed as an average of duplicates/triplicates ± SEM. iMark microplate reader and software was used for data analysis (Bio-Rad Laboratories Inc., Hercules, CA, USA).

### ELISA for detection of MOG Ab

96-well plates were coated with 10 µg/ml conformational mMOG or conformational hMOG in 1xPBS overnight. Diluted samples were added for 2 h. After washing, plate-bound Ab of murine samples and 8.18C5 Ab were detected with horseradish peroxidase (HRP)-conjugated anti-mouse IgG, directed against the Fc part of the bound Ab (1:6000; Sigma-Aldrich) or against the whole molecule (1:5000; Sigma-Aldrich). Anti-MOG Ab in human samples were detected with HRP-conjugated anti-human IgG (1:1000; Sigma-Aldrich). Plates were read at 450 nm wavelength by a Tecan Genios plate reader and analyzed using Magellan6 software or iMark microplate reader and software.

### Histology

Brain and spinal cord tissue was PBS-perfused and cryofixed or perfused and fixed with 4 % paraformaldehyde and paraffin embedded. To determine myelin loss and inflammatory infiltration, vertically or horizontally oriented sections were stained with Luxol Fast Blue and periodic acid Schiff (LFB/PAS) or hematoxylin and eosin (H&E), respectively. Infiltration of immune cells was determined by immunohistochemistry using Ab against CD3 (1:150 dilution; DCS, Hamburg, Germany), B220 (1:200 dilution; BD Pharmingen) or MAC3 (1:200 dilution, BD Bioscience). Evaluation of histologic samples was performed in a blinded manner.

### Statistical analysis

EAE experiments were evaluated for significance using the Mann–Whitney test. The Student *t* test was used for all other statistical comparison. A value of *p* < 0.05 was considered significant. Data are presented as mean ± SEM.

## Results

### Depletion of CD20 positive B cells does not alter severity of induced EAE in Th mice nor spontaneous EAE development in Thx2D2 mice

**Fig. 1:**
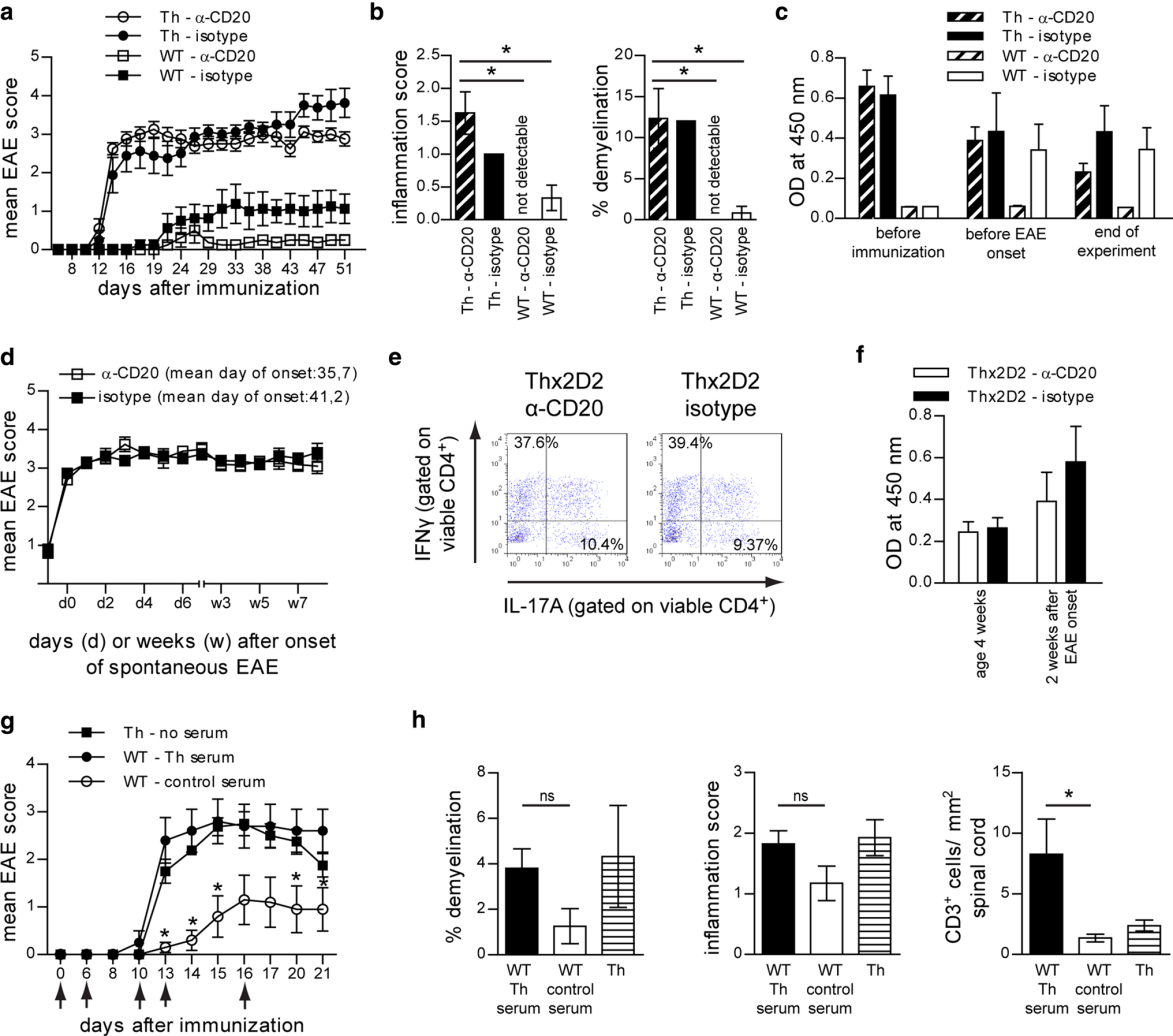
B cells are not required for fulminant induced EAE in Th mice nor for spontaneous EAE in Thx2D2. **a**–**c** Th or WT mice were treated weekly with anti (α)-CD20 or isotype control Ab starting 3 weeks prior to immunization with mMOG. **a** Mean group EAE score ± SEM; *n* = 10 mice/group; * = *p* < 0.05 (Mann–Whitney; data represent five independent experiments). **b** CNS inflammatory damage at day 48 after immunization. Indicated is the mean group score ± SEM of spinal cord inflammation (H&E; *left*), determined as: 0 = no inflammation, 1 = slight inflammation. 2 = moderate inflammation, 3 = strong inflammation and the mean % ± SEM of demyelinated spinal cord area per group (LFB/PAS; right); *n* = 3–4 mice/group; * = *p* < 0.05 (Mann–Whitney). **c** Anti-mMOG IgG Ab serum levels determined by ELISA (dilution 1:40,500); *n* = 10 mice/group. **d**–**f** Thx2D2 mice were treated weekly with anti (α)-CD20 or isotype control Ab starting 28 days after birth. **d** Mean group score and mean day of onset of spontaneous EAE in anti (α)-CD20 (*n* = 18) and isotype Ab treated mice (*n* = 22) ± SEM; *p* = ns (Mann–Whitney). **e** Frequency of IFN-γ and IL-17A producing T cells isolated from the CNS at the end of experiment. **f** Anti-mMOG IgG Ab determined by ELISA (serum dilution 1:13,500); *n* = 6–16 mice/group. **g**, **h** EAE was induced in WT mice by immunization with MOG p35-55. *Arrows* indicate i.v. injections of serum containing pathogenic anti-mMOG Ab (Th serum) or non-pathogenic Ab (control serum). Immunized Th mice not receiving any serum, served as positive control. **g** Mean group EAE score ± SEM; *n* = 8–10 mice/group; * = *p* < 0.05 (WT Th serum recipients vs. WT control; Mann–Whitney; data represent three independent experiments). **h** CNS inflammatory damage at day 25 after immunization. Indicated is the mean % ± SEM of demyelinated spinal cord white matter area per group (LFB/PAS; *left*), the mean group score ± SEM of inflammation (H&E; *middle*), determined as follows: 0 = no inflammation, 1 = slight inflammation. 2 = moderate inflammation, 3 = strong inflammation and the number of T cells/mm^2^ spinal cord ± SEM per group determined by CD3^+^ immunostaining (*right*); *n* = 4 mice/group; * = *p* < 0.05 (Mann–Whitney)

To delineate the relative pathogenic contribution of myelin-specific Ab from myelin-specific B cells, we first investigated the effect of preventive B cell depletion on actively induced EAE in Th mice constitutively containing a high frequency of MOG-recognizing B cells as well as plasma cells producing pathogenic anti-MOG Ab (Suppl. Figure 1a). Results were compared to WT mice, in which only a comparably small fraction of B cells can respond to mouse MOG protein immunization, and developing Ab titers are non-pathogenic [32]. As shown in suppl. Figure 1b, three injections of anti-mouse CD20 Ab efficiently depleted virtually all CD20 positive (^+^) B cells from blood, bone marrow, lymph node and spleen of WT mice, sparing only CD20 negative B cells in the bone marrow. Corresponding results were obtained in secondary lymphoid organs of Th mice following anti-CD20 Ab treatment (Suppl. Figure 1c). Upon immunization with a suboptimal dose of mMOG protein, WT mice developed mild but consistent EAE with a slight benefit in the B cell-depleted group [52]; using the identical induction regimen, Th mice developed fulminant EAE irrespective of anti-CD20 Ab treatment prior to immunization (Fig. 1a) or after EAE onset (Suppl. Figure 1d). These findings were closely reflected by a massive CNS demyelination and inflammation in both B cell-depleted and isotype-treated Th mice (Fig. 1b), along with an unhindered Th1/Th17 differentiation in the absence of B cells (Suppl. Figure 1e). In line with the fact that anti-CD20 Ab spare CD20 negative plasma cells, constitutively produced anti-MOG Ab remained high in preventatively anti-CD20 Ab treated Th mice during the priming- and CNS-infiltration period (Fig. 1c).

Next, we applied anti-CD20 Ab treatment to Thx2D2 mice (Suppl. Figure 1f) containing MOG-recognizing B cells, MOG-specific T cells and high titers of constitutively produced pathogenic anti-MOG Ab. To our surprise, preventative B cell depletion at the age of 3–4 weeks neither affected incidence of spontaneous EAE (18 anti-CD20-treated vs. 22 isotype-treated in equally sized groups) nor its severity (Fig. 1d). As in immunized Th mice, depletion of CD20^+^ B cells did not affect generation of encephalitogenic Th1 and Th17 cells (Fig. 1e) nor lower endogenous anti-MOG Ab production at the time of disease induction (Fig. 1f); analogous clinical and immunological results were obtained when anti-CD20 Ab treatment was initiated in fully established spontaneous EAE (Suppl. Figure 1g–h).

### Transfer of serum from Th mice exacerbates experimental autoimmune encephalomyelitis in wild-type mice

These findings endorsed a prominent pathogenic role of myelin-reactive Ab independent of B cells, which we next investigated directly and independent of anti-CD20 Ab treatment by adoptive transfer. At first, serum from immunized Th mice (Th serum) was transferred into MOG p35-55-immunized WT mice. As shown in Fig. 1g, repeated intravenous (i.v.) injections of Th serum indeed substantially accelerated EAE severity in WT recipients and, of note, onset and severity closely resembled EAE in immunized Th mice. Histologic analysis revealed accentuated CNS inflammation and demyelination in Th serum recipients (Fig. 1h, left and middle), which is in line with the reported demyelinating nature of anti-MOG Ab in ongoing EAE [26, 46]. Exceeding this anticipated result, Th serum recipients furthermore displayed a massive CNS infiltration of CD3^+^ T cells (Fig. 1h, right).

### Transfer of anti-MOG antibodies triggers spontaneous experimental autoimmune encephalomyelitis in MOG T cell receptor transgenic 2D2 mice

**Fig. 2:**
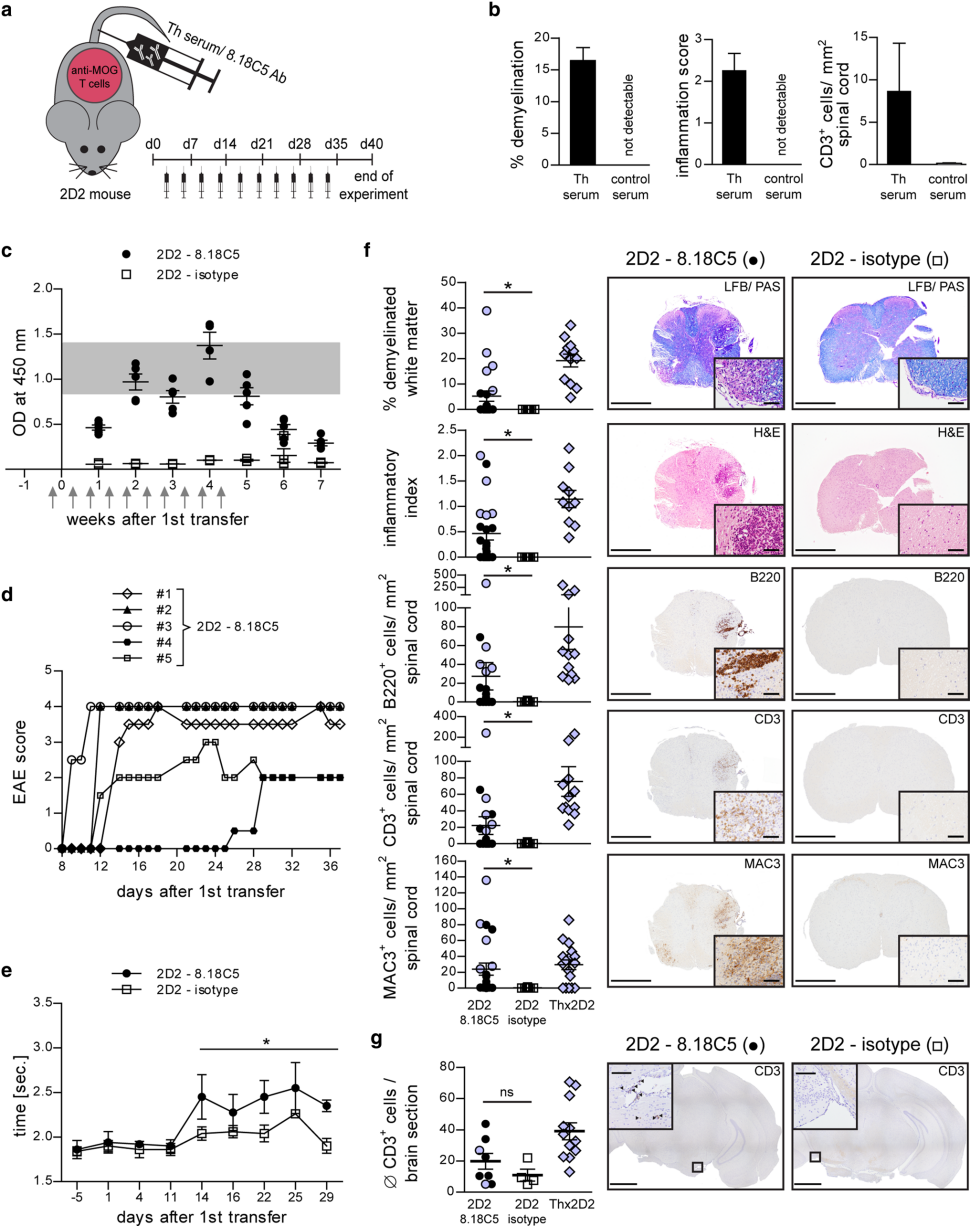
Transfer of anti-mMOG Ab containing serum or purified anti-mMOG Ab triggers spontaneous EAE in 2D2 recipients. **a** Overview of experimental setup. **b** Naïve 2D2 mice received i.v. injections of Th serum or control serum. CNS inflammatory damage determined at day 18 after EAE onset. Indicated is the mean % ± SEM of demyelinated spinal cord area per group (LFB/PAS; *left*), the mean group score ± SEM of spinal cord inflammation (H&E; *middle*), determined as follows: 0 = no inflammation, 1 = slight inflammation. 2 = moderate inflammation, 3 = strong inflammation and the number of T cells/mm^2^ spinal cord ± SEM per group determined by CD3^+^ immunostaining (*right*); *n* = 2 mice/group. **c**–**g** Naïve 2D2 mice received i.v. injections of 8.18C5 or isotype control Ab. **c** Anti-mMOG Ab serum levels in recipients determined by ELISA (dilution 1:13,500). *Arrows* indicate injections of 8.18C5 or isotype control Ab. *Gray area* represents the range of anti-mMOG Ab predetermined in spontaneously EAE-diseased Thx2D2 mice; *n* = 5 mice/group. **d** Individual EAE courses of 2D2 mice receiving 8.18C5 Ab. **e** Elevated beam test of 2D2 mice receiving 8.18C5 or isotype control Ab. Indicated is the mean time in seconds (sec.) per group ± SEM required to traverse the beam; *n* = 4–5 mice/group; * = *p* < 0.05 (Mann–Whitney). **f** Demyelination (LFB/PAS), overall inflammation (H&E) and immune cell infiltration of the spinal cord evaluated by CD3-, B220- and MAC3-immunohistochemistry 40 days after first Ab transfer. Representative sections (*right*) and group mean % ± SEM of demyelinated white matter area, number of inflammatory spots per spinal cord section (inflammatory index) and of infiltrating immune cells/mm^2^ spinal cord (*left*). Mice with paralysis in *purple*; for comparison, Thx2D2 mice with EAE are depicted. *Scale bar* overview = 500 µm, *scale bar* inlay = 50 µm; *n* = 12–23 mice/group; * = *p* < 0.05 (Mann–Whitney). **g** CD3 immunostaining of T cells in the brain at day 40 after first transfer. Representative sections (*right*) and absolute number of infiltrating T cells/section (*left*). Mice with paralysis in purple; for comparison Thx2D2 mice with EAE are depicted. *Scale bar* overview = 1000 µm, *scale bar* inlay = 100 µm; *n* = 4–12 mice/group; *p* = ns (Mann–Whitney)

**Fig. 3:**
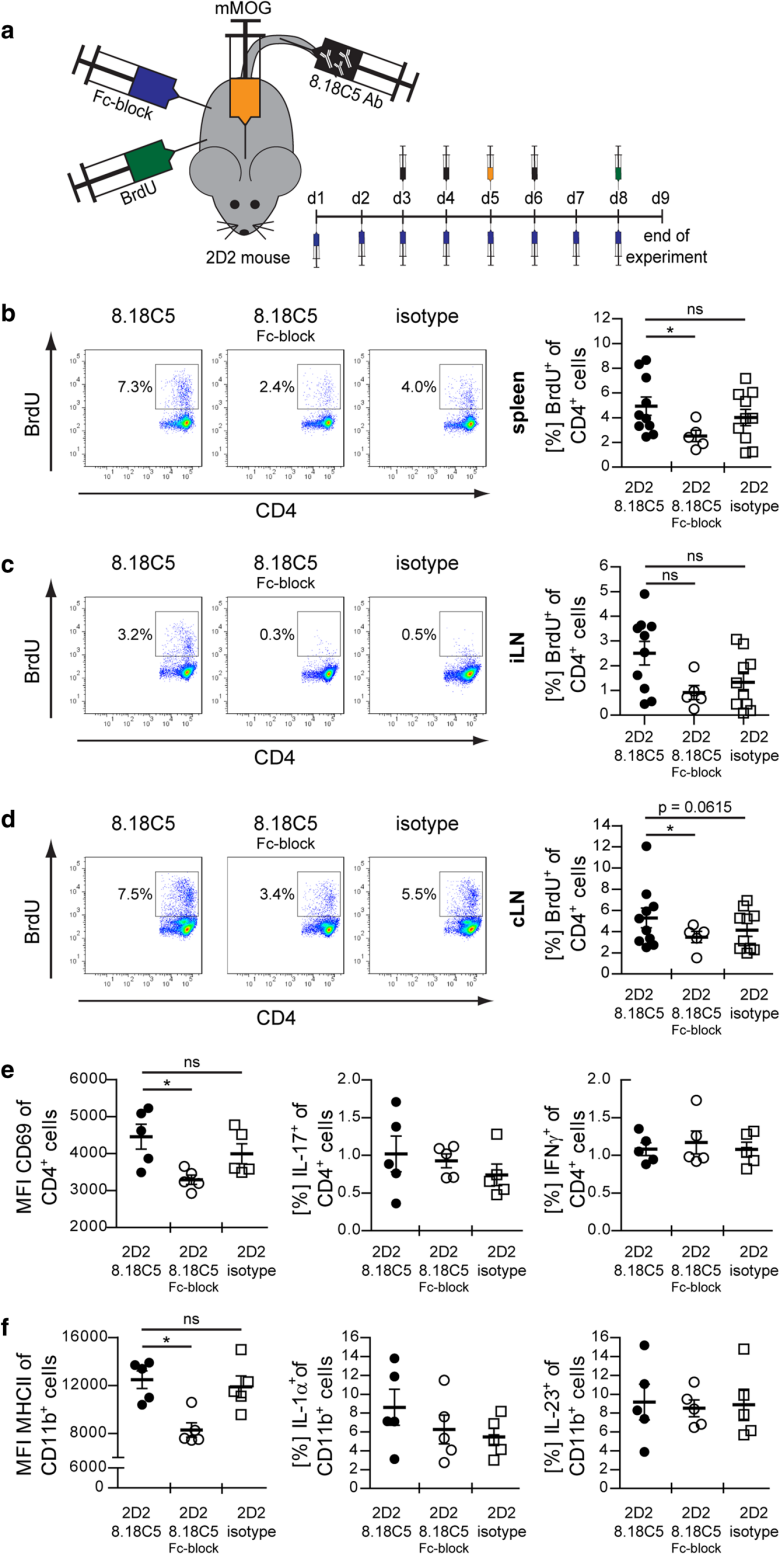
Transfer of anti-mMOG Ab into 2D2 mice triggers in vivo activation and expansion of peripheral myelin-reactive T cells in an Fc-dependent manner. **a** Overview of experimental setup. **a**–**f** Naïve 2D2 mice were treated daily with Fc receptor blocking anti-CD16/CD32 or control Ab i.p. during the entire experiment. Starting at day (d)3, mice received three consecutive i.v. injections of 8.18C5 or control Ab. mMOG was injected intrathecally at d5. 24 h before evaluation, mice received BrdU i.p. (d8). In vivo proliferation of CD4^+^ T cells (pre-gated on viable CD4^+^ cells) analyzed by BrdU uptake in **b** spleen, **c** inguinal lymph nodes (iLN) and **d** cervical lymph nodes (cLN). **b**–**d** Representative FACS plots (*left*) and frequency of BrdU^+^ CD4^+^ cells in % ± SEM (*right*); *n* = 5–10 mice/group; * = *p* < 0.05 (*t* test). **e** Expression of CD69 (mean fluorescence intensity; MFI) and % of IL-17A and IFN-γ producing CD4^+^ cells ± SEM isolated from cLN (pre-gated on intact cells). **f** Expression of MHC class II (MHCII; MFI) and % of IL-1α and IL-23 producing CD11b^+^ cells ± SEM isolated from cLN (pre-gated on intact cells)

Together with the observation that early B cell depletion failed to halt spontaneous EAE development in Thx2D2 mice, these findings fueled our hypothesis that in lieu of antigen-specific B cells, anti-MOG Ab may be capable of triggering activation and expansion of myelin-reactive T cells in a manner sufficient to cause EAE. To test this hypothesis, we transferred serum from Th mice or purified 8.18C5 Ab into otherwise naïve 2D2 recipients, which contain MOG-specific T cells but no transgenic B cells (Fig. 2a). Strikingly, five out of fourteen 2D2 mice receiving Th serum developed classical EAE with ascending paralysis, one mouse developed atypical EAE with severe ataxia (Table 1). Histologic analysis confirmed that all EAE-diseased Th serum recipients showed extensive CNS demyelination, inflammation and infiltration by CD3^+^ T cells (Fig. 2b), while none of the twelve 2D2 mice receiving control serum showed signs of clinical or histological EAE. To confirm that myelin-reactive Ab and not any other serum component had triggered EAE, we next transferred 150 µg of purified 8.18C5 Ab, the amount equivalent to its content in 150 µl Th serum. As indicated in Fig. 2c, 2D2 mice receiving a total of 10 injections showed a rapid increase in anti-MOG Ab, comparable to the level observed in Thx2D2 mice with spontaneous EAE (gray area). Again, thirteen out of twenty-three 8.18C5 Ab recipients, but none of the seventeen 2D2 mice receiving control Ab, developed severe clinical or histologic EAE (Table 1; Fig. 2d). When evaluated by the more sensitive elevated beam test, 8.18C5 Ab recipients without paralysis yet showed a significant decline in their agility (Table 1; Fig. 2e). CNS damage was quantified in all 8.18C5 Ab-transferred 2D2 mice, which revealed a pronounced inflammation and CNS infiltration by both B and T cells in the majority of these mice, while extensive demyelination was restricted to the paralyzed subgroup (Fig. 2f + g). Here, all histologic parameters were in their extent comparable to Thx2D2 mice with spontaneous fulminant EAE, while in some cases, the degree of infiltration by highly activated MAC3^+^ macrophages even exceeded the level in diseased Thx2D2 mice. To investigate in which sequence peripherally applied anti-MOG Ab, MOG-reactive T cells and activated myeloid APC may have triggered EAE, we transferred anti-MOG Ab into naïve WT mice (Suppl. Figure 2a). Recipients showed no signs of paralysis, impaired agility or, importantly, CNS demyelination (Suppl. Table 1; Suppl. Figure 2b + c), suggesting that in the absence of ongoing CNS inflammation, myelin within the CNS is not recognized by peripherally applied myelin-reactive Ab. Alternatively, myelin-reactive Ab could have triggered activation of peripheral myelin-reactive T cells prior to CNS entry. To investigate this hypothesis, we evaluated proliferation of T cells in lymph nodes and spleen of healthy, naïve 2D2 mice after peripheral injection of 8.18C5 Ab and co-administration of mMOG protein intrathecally (Fig. 3a; Suppl. Figure 3a + c). As shown in Fig. 3b–d, 8.18C5 Ab triggered in vivo expansion of CD4^+^ T cells in spleen, inguinal and cervical lymph nodes 4 days after mMOG injection and 6 days after the initial administration of 8.18C5 Ab. While at this early time point only a trend toward an enhanced Th17 differentiation could be observed, administration of 8.18C5 Ab lead to a distinct upregulation of the early activation marker CD69 on CD4^+^ T cells (Fig. 3e). Further, co-administration of 8.18C5 Ab and mMOG was associated with a concomitant upregulation of MHC class II and an enhanced release of IL-1α by CD11b^+^ myeloid APC (Fig. 3f). To a lesser extent, T cell proliferation and activation of myeloid APC were also observed in 2D2 mice receiving control Ab, which may be reflective of endogenous anti-MOG Ab development in these mice when MOG is administered intrathecally. Irrespectively, 8.18C5 Ab-mediated in vivo activation and proliferation of T cells as well as activation of myeloid APC could be completely abolished by addition of an in vivo Fc-blocking Ab (Suppl. Figure 3b; Fig. 3b–f). In conjunction, these findings indicate that myelin-specific Ab trigger activation of peripheral myelin-specific T cells and highlight Fc-mediated opsonization of antigen as most plausible mechanism.

**Table 1:** Summary of clinical and histological results upon transfer of anti-MOG Ab containing serum or of purified anti-mMOG Ab into naïve 2D2 mice

	Animals with histological or clinical signs of EAE				Animals with impaired agility^a^
	Number of animals with CNS inflammation	Number of animals with clinical symptoms	Mean max. EAE severity (range)	Onset of EAE; mean days after 1st serum/Ab transfer (range)	Number of mice with ≥25 % deterioration in agility testing; measured by increase in time
2D2—Th serum	6/14	6/14	3.1 (2.5–3.5)	12.8 (5–24)	n.a.
2D2—control serum	0/12	0/12	n.a.	n.a.	n.a.
2D2—8.18C5	13/23	5/23	3.4 (2.0–4.0)	14.2 (9–26)	6/8
2D2—isotype control	0/17	0/17	n.a.	n.a.	2/9

^a^Only mice without signs of classical EAE (paralysis) tested

### Anti-MOG antibodies promote low dose recognition of MOG by myeloid antigen-presenting cells permitting activation of MOG-reactive T cells

**Fig. 4:**
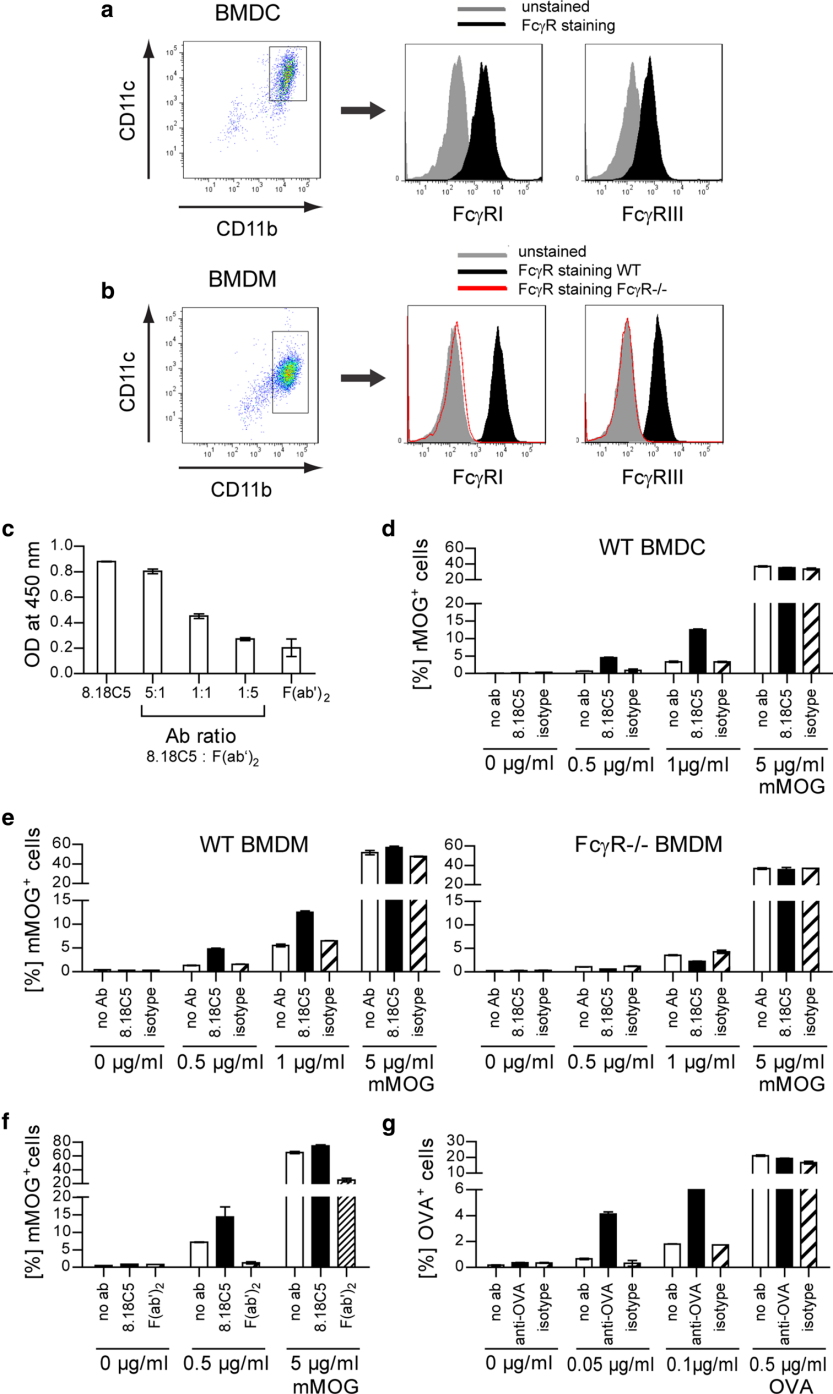
Opsonization of conformational mMOG by anti-mMOG Ab fosters Fc-mediated antigen uptake. Mean expression of Fcγ receptor (FcγR)I and FcγRIII by **a** WT BMDC and **b** WT or FcγR^−/−^ BMDM. **c** Competitive binding of 8.18C5 F(ab′)_2_ and intact 8.18C5 Ab at various ratios to mMOG; plate-bound Ab detected by anti-mouse IgG Ab against the Fc part. **d**–**f** Phagocytosis of mMOG-DyLight-405 by APC. APC were incubated with mMOG-DyLight-405 in the presence of 8.18C5, or isotype control Ab (mean % of mMOG-DyLight-405 positive (mMOG^+^) APC ± SEM, gated on intact CD11b^+^/CD11c^+^ cells). **d** Phagocytosis of mMOG by WT BMDC. Representative data set shown; combined statistical analysis of two independent experiments: *p* < 0.05 for 8.18C5 vs. isotype Ab at 0.5 and 1 μg/ml mMOG (*t* test). **e** Phagocytosis of mMOG by WT (*left*) or FcγR^−/−^ (*right*) BMDM. Representative data set shown; combined statistical analysis of two independent experiments: *p* < 0.05 for 8.18C5 vs. isotype control Ab at 0.5, 1 and 5 μg/ml mMOG of WT BMDM (*t* test). **f** Phagocytosis of mMOG by WT BMDM, additionally in the presence of 8.18C5 F(ab′)_2_ fragments. Representative data set shown; combined statistical analysis of two independent experiments: *p* < 0.05 for 8.18C5 Ab vs. 8.18C5 F(ab′)_2_ at 0.5 and 5 μg/ml mMOG (*t* test). **g** Phagocytosis of OVA-FITC by BMDM. BMDM were incubated with OVA-FITC in the presence of anti-OVA Ab or isotype control Ab (mean % of OVA-FITC positive (OVA^+^) BMDM, gated on intact CD11b^+^/CD11c^+^ cells). Representative data set shown; combined statistical analysis of two independent experiments: *p* < 0.05 for anti-OVA vs. isotype control Ab at 0.05 and 0.1 μg/ml OVA (*t* test)

Activation of CD4^+^ T cells requires antigen presentation in the context of MHC class II. Prior to its presentation, antigen must be taken up and presented by the immune system, which, particularly at low concentrations, can be facilitated by specific binding to B cells [9, 17, 38] or Ab [22, 31]. In the latter case, Ab-decoration triggers Fc receptor-mediated internalization and processing of the antigen by APC [49], followed by its presentation to adaptive immune cells. This mechanism of Ab-mediated antigen opsonization is believed to be central for instance in development of systemic lupus erythematosus (SLE) [15]. To investigate whether in our setting, MOG-specific Ab may have triggered in vivo activation of myelin-specific T cells [35] by opsonization of rare CNS auto-antigen, we developed an in vitro setting capable of dissecting the respective components; we generated BMDM or BMDC from WT mice expressing high levels of Fc receptors or from knockout mice lacking Fcγ receptors (FcγR^−/−^) (Fig. 4a + b). Further, we digested the 8.18C5 Ab to obtain an antigen-recognizing, but Fc-cleaved F(ab′)_2_ fragment (Fig. 4c; Suppl. Figure 4a + b). Using these tools, we demonstrated that intact 8.18C5 Ab strongly promoted recognition and internalization of fluorescence-labeled mMOG by either BMDC or BMDM (Fig. 4d + e, left). This effect strictly depended on cellular expression of Fcγ receptors (Fig. 4e, right); along the same lines, the Fc-cleaved 8.18C5 F(ab′)_2_ fragment failed to enhance internalization and rather neutralized antigen recognition (Fig. 4f). Of interest, Ab-mediated opsonization similarly occurred when the random control antigen OVA was used in combination with anti-OVA Ab (Fig. 4g), corroborating that Ab opsonize any fitting antigen.

**Fig. 5:**
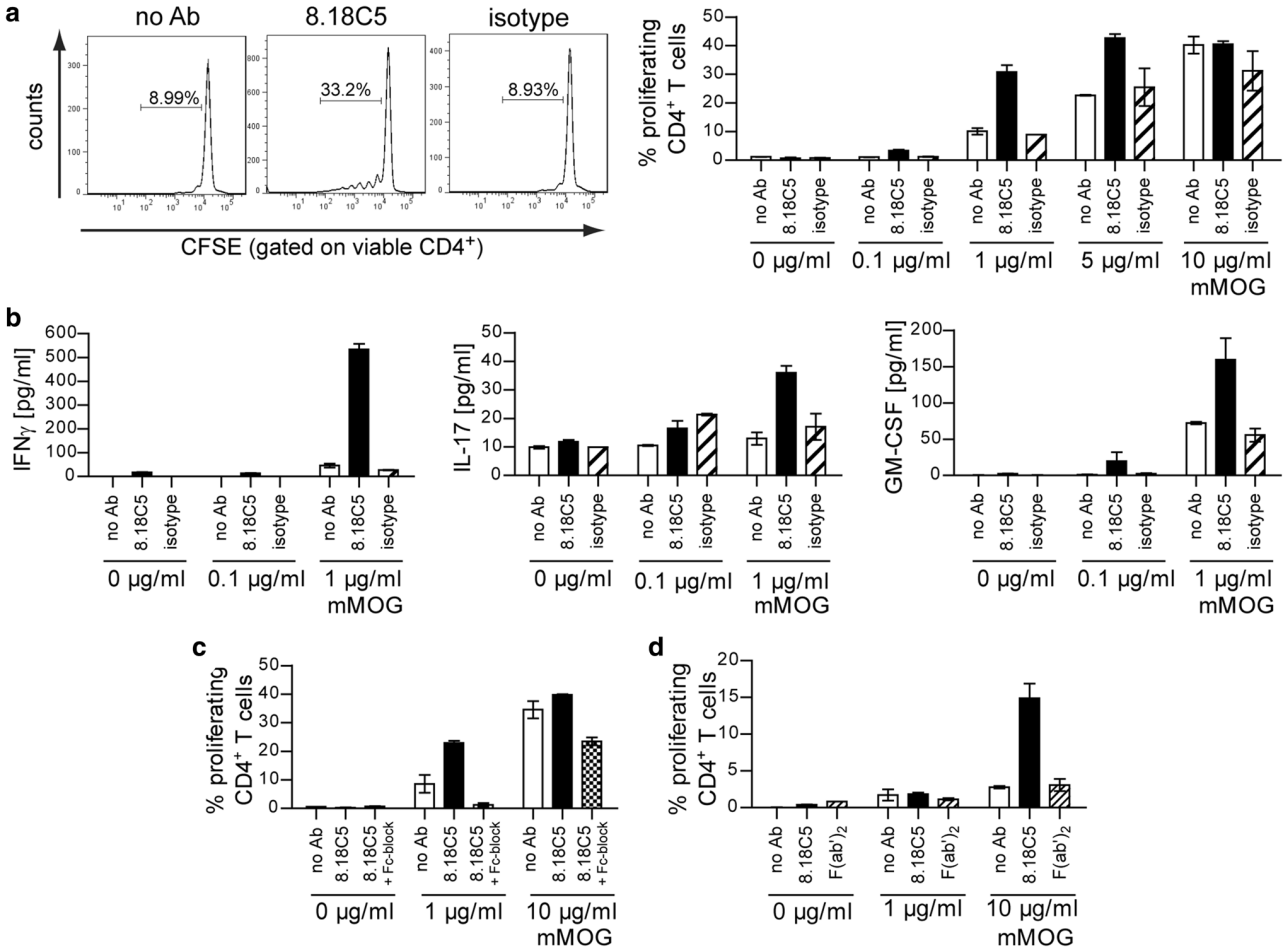
Opsonization-triggered antigen uptake results in an increased capability of myeloid APC to generate encephalitogenic T cells. **a**–**d** WT BMDM co-cultured with CFSE-labeled MOG-specific 2D2 T cells in the presence of mMOG. **a** Proliferation of CD4^+^ T cells in the presence of 8.18C5 or isotype control Ab determined by CFSE dilution (representative FACS plot, *left*; mean % of proliferating T cells in duplicates ± SEM, *right*). Representative data set shown; combined statistical analysis of four independent experiments: *p* < 0.05 for 8.18C5 vs. isotype control Ab at 0.1 μg/ml mMOG (*t* test). **b** Differentiation of naïve T cells into Th1-, Th17- or GM-CSF-producing T cells determined by production of IFN-γ, IL-17 or GM-CSF in the presence of 8.18C5 or isotype control Ab (duplicates ± SEM). **c** Proliferation of CD4^+^ T cells in the presence of 8.18C5 Ab or a combination of 8.18C5 Ab and Fcγ receptor blocking anti-CD16/CD32 Ab (8.18C5 + Fc-block) determined by CFSE dilution (mean % of proliferating T cells in duplicates ± SEM). Representative data set shown; combined statistical analysis of two independent experiments: *p* < 0.05 for 8.18C5 Ab vs. 8.18C5 F(ab′)_2_ at 1 μg/ml mMOG and *p* = 0.054 at 10 µg/ml mMOG (*t* test). **d** Proliferation of CD4^+^ T cells in the presence of 8.18C5 Ab or the 8.18C5 F(ab′)_2_ fragment determined by CFSE dilution (mean % of proliferating T cells in duplicates ± SEM)

To understand the downstream immunological consequences of Ab-mediated antigen opsonization we co-cultured WT BMDM with naïve 2D2 T cells, again in the presence of mMOG. In line with our in vivo findings, addition of 8.18C5 Ab specifically accelerated T cell proliferation and differentiation into encephalitogenic T cells, while blockade of Fcγ receptors on APC reversed this effect (Fig. 5a–c; Suppl. Figure 4c). Along the same lines, the 8.18C5 F(ab′)_2_ fragment again failed to enhance internalization and subsequent presentation of mMOG to myelin-specific T cells (Fig. 5d). In summary, these results indicate that in development of CNS autoimmune disease, systemic auto-Ab facilitate APC recognition of otherwise undetected CNS antigen, resulting in activation, pro-inflammatory differentiation and CNS infiltration of auto-reactive T cells.

### Patient-derived anti-MOG antibodies mediate opsonization of human MOG protein

**Fig. 6:**
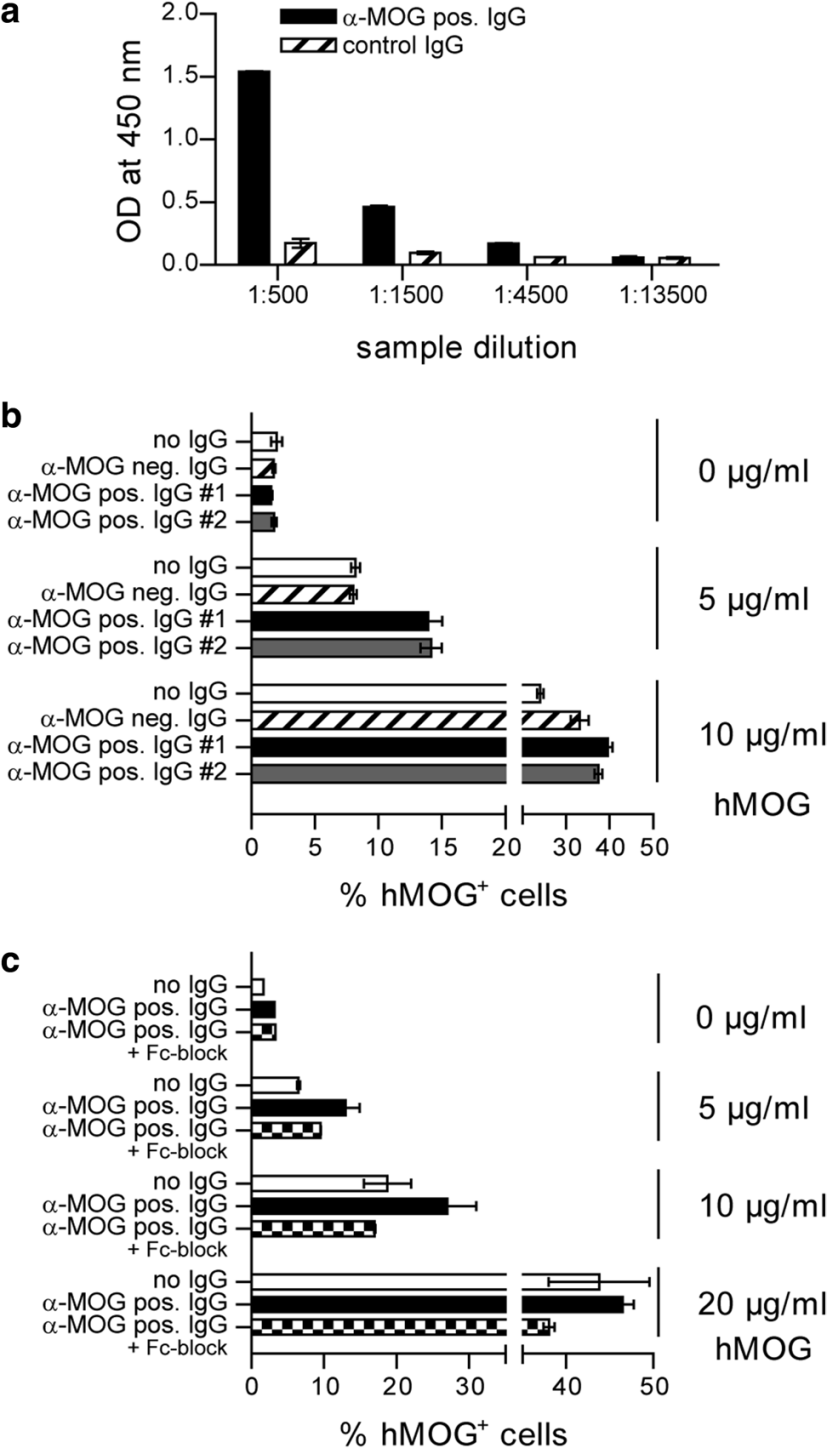
IgG isolated from patients with NMOSD facilitate recognition of hMOG protein. **a** Detection of Ab against hMOG in a purified IgG fraction of a patient with NMOSD (α-MOG pos. IgG) by ELISA. Plate-bound Ab were detected with anti-human IgG Ab directed against the Fc part. For ELISA, intravenous IgG (control IgG) served as negative control. **b** Phagocytosis of hMOG-DyLight-405 by WT BMDM. Cells were incubated with hMOG-DyLight-405 in the presence of IgG samples from NMOSD patients containing anti-MOG Ab (α-MOG pos. IgG #1 and #2) or an anti (α)-MOG Ab negative (neg.) IgG preparation from a healthy individual (mean % of hMOG-DyLight-405 positive (hMOG^+^) APC, gated on intact CD11b^+^/CD11c^+^ cells). Representative data set shown; *p* < 0.05 for α-MOG pos. IgG #1 vs. α-MOG neg. IgG and α-MOG pos. IgG #2 vs. α-MOG neg. IgG at 5 µg/ml hMOG (*t* test). **c** Phagocytosis of hMOG-DyLight-405 by WT BMDM. Cells were incubated with hMOG-DyLight-405 in the presence of anti-MOG positive IgG #2 or a combination of anti-MOG positive IgG #2 and Fcγ receptor blocking anti-CD16/CD32 Ab (8.18C5 + Fc-block; mean % of hMOG^+^ APC, gated on intact CD11b^+^/CD11c^+^ cells)

To exemplify that human IgG is capable of opsonizing human myelin protein, we next investigated IgG preparations obtained from two NMOSD patients with high titers of anti-MOG Ab determined in an established cell-based assay [30]. As a prerequisite to be tested in our in vitro assay, we first ensured that patient-derived anti-MOG Ab recognized soluble hMOG in vitro by a conventional ELISA (Fig. 6a). Applying the IgG preparations to our phagocytosis assay, we indeed observed that both anti-MOG Ab containing IgG preparations significantly enhanced the APC uptake of hMOG, when compared to an anti-MOG Ab negative IgG preparation obtained from a healthy individual (Fig. 6b). Ab-mediated opsonization could be largely reversed by addition of an Fc receptor blocking Ab (Fig. 6c), mechanistically corroborating that under these conditions human anti-MOG Ab facilitate APC uptake of human CNS antigen in an Fc-dependent manner.

## Discussion

Antigen-specific B cells are increasingly recognized to act as important APC in models of human CNS demyelinating diseases. High affinity BCR binding of antigen triggers B cell activation and enables subsequent presentation of processed antigen to T cells. Thus, when B cells and T cells share antigen recognition, B cells are the dominant APC population and mere co-existence of antigen-specific B and T cells can prompt spontaneous CNS autoimmune disease [3, 21, 39]. A recent publication demonstrated that in this setting, spontaneous EAE can occur without the requirement of myelin-specific Ab [34]. Here, we investigated the inverse scenario, and report that in the absence of antigen-specific B cells, myelin-specific Ab in conjunction with T cells recognizing MOG suffice to trigger spontaneous EAE. At first sight, these two reports on the relevance of B cells versus myelin-specific Ab appear conflicting; upon closer view though, the link between both observations is specific recognition of endogenously rare antigen, which can be achieved by two distinct, yet related mechanisms: by binding to the BCR of B cells or alternatively to the corresponding Ab, which permits Fc-mediated antigen recognition by myeloid APC. Both mechanisms result in antigen uptake, processing and presentation by B cells or myeloid APC, respectively. This concept is supported by several mechanistic observations; in Thx2D2 mice, very low concentrations of mMOG added to a single cell suspension of purified splenocytes stimulated T cell proliferation [3, 21], while this effect was abolished when B cells were removed. Correspondingly, in our study, the addition of MOG-specific Ab enabled myeloid APC to recognize antigen at very low concentrations in an Fc-dependent manner and to activate co-cultured T cells. Antigen-specific B cells and antigen-specific Ab may thus independently contribute to the risk to develop CNS autoimmune disease, while the common element of disease initiation is specific recognition of rare CNS antigen subsequently boosting a disease-driving auto-reactive T cell response.

In line with this concept, we could show that anti-CD20 Ab-mediated depletion of myelin-specific B cells did not affect development of encephalitogenic T cells and failed to prevent or ameliorate EAE in the presence of endogenously produced myelin-specific Ab. Transfer of serum obtained from Th mice containing high levels of anti-MOG Ab exacerbated EAE in WT recipients in an extent indistinguishable from the fulminant course in Th mice containing both myelin-specific Ab and B cells. Furthermore, Th serum or purified anti-MOG Ab triggered spontaneous EAE in naïve 2D2 recipients. In conjunction, these findings evidently support a crucial role of myelin-specific Ab independent of myelin-specific B cells, which is further corroborated by the observation that in the absence of an immediate clinical benefit, Th mice receiving anti-CD20 Ab either in prevention or reversal showed a slight amelioration late in the chronic course of EAE, when levels of myelin-specific Ab declined (Fig. 1a + c; Suppl. Figure 1d). It is unclear and rightful to question whether in patients with MS, endogenous self-reactive Ab titers can reach corresponding functional relevance. Nevertheless, it could be important to recognize the general concept that Ab-mediated opsonization can functionally bypass antigen-specific B cells and that this alternative pathway of self-antigen recognition is not targeted and likely not affected by the soon to be approved MS treatment of anti-CD20 Ab. Accordingly, Ab-mediated antigen recognition by myeloid APC may become particularly important when antigen-specific B cells are extinguished, a notion to be kept in mind when individuals fail to respond to anti-CD20 Ab treatment [2, 7].

One central and widely unanswered question in the pathogenesis of CNS autoimmune disease remains where and how CNS antigens are initially recognized. In the absence of ongoing inflammation, the CNS is a well-protected, immune-privileged site and molecularly large Ab should not be capable of crossing the intact blood–brain barrier in a meaningful manner. Therefore, it is rather unlikely that peripherally administered myelin-reactive Ab can recognize and possibly degrade intact myelin within the otherwise healthy CNS. One of our control settings, in which anti-MOG Ab were transferred into naïve WT mice, consolidates this notion as despite intensive investigation, no CNS damage was observed. Alternatively, a plausible site of initial recognition of CNS antigen is cervical lymph nodes [10, 40], where vessels drain brain interstitial fluid [1, 28]. Supporting this concept, traces of myelin have been detected in CNS draining deep cervical lymph nodes of patients with MS [13]. A paralleling observation was reported in EAE-diseased mice [53], and surgical excision of cervical lymph nodes reduced relapse severity in chronic EAE [50]. To corroborate this possible path of CNS drainage we first injected 10 % Evans blue intrathecally and indeed retrieved the blue dye 1 h after its injection from cervical lymph nodes (suppl. Figure 3c). We next applied MOG protein intrathecally and in addition injected 8.18C5 Ab peripherally into healthy, naïve 2D2 recipients. Four days after MOG injection and 6 days after initial application of anti-MOG Ab, this regimen caused a marked in vivo expansion of CD4^+^ T cells in lymph nodes and spleen of otherwise healthy mice. In conjunction, these data point toward peripheral lymphoid organs as the most plausible site where CNS-reactive Ab facilitated initial recognition of CNS-drained antigen, which subsequently triggered development of an encephalitogenic T cell response.

Using human IgG preparations in our in vitro setting was meant to exemplify that peripherally produced human anti-CNS Ab are generally capable of opsonizing human CNS antigen. To date, it remains unknown whether this effect occurs in patients with CNS demyelinating disorders in a functionally meaningful manner, although early development of anti-MOG Ab in MS [23] as well as the pronounced peripheral production of Ab against AQP-4 or MOG in NMO and NMOSD patients [20] highlights this possibility. Ab-decoration of auto-antigen is indeed known to occur in a variety of paraneoplastic disorders [44] and to enhance severity of systemic autoimmune conditions, such as SLE [42, 47]. In its pathogenesis, Ab directed against nuclear antigens are believed to opsonize apoptotic cells enhancing their uptake by dendritic cells [14], which in return may augment an autoimmune T cell response [15]. Further paralleling our findings in patients in an intriguing manner, serum from SLE patients specifically enhanced in vitro phagocytosis of apoptotic Jurkat cells by normal healthy donor macrophages [45]. The clinical relevance of this effect is further supported by the observation that the effectiveness of anti-CD20 Ab treatment negatively correlates with an expanded auto-Ab profile in patients with SLE [6]. Besides inducing or promoting autoimmunity, the general concept of Ab-mediated opsonization can also be applied therapeutically, when monoclonal Ab are used to alert the immune system to tumor cells enhancing cell-mediated cytotoxicity [43] as well as priming of an adaptive immune response [48]. Lastly, immunotherapy via Ab-mediated opsonization is currently pioneered to target pathological protein deposits in treatment of neurodegenerative disorders [5, 33]. In summary, Ab-mediated opsonization widely occurs in development and progression of systemic autoimmune conditions and is therapeutically harnessed to enhance clearance of potentially harmful antigen.

To date, the role of self-reactive Ab in MS has been primarily projected into enhancing CNS demyelination in ongoing acute disease flares [16], while in NMO, anti-AQP-4 Ab are believed to selectively target astrocytes, followed by severe secondary demyelination [29]. Our findings reported here extend this view and suggest that in addition, CNS-reactive Ab may be crucial for initiating and amplifying an adaptive autoimmune response to traces of otherwise unrecognized CNS auto-antigen. This novel concept has several important implications: first, while CNS-reactive Ab may initiate this chain of events, they require responding T cells to hereby cause harm. This may explain why myelin-specific Ab can also be found in controls and healthy volunteers, which do not develop CNS demyelination [23]. Second, in the context of MS, a serum auto-Ab response, particularly against MOG, widely occurs at early disease stages and in children with MS [11, 23]. In light of our new findings and opposite to our current understanding, this humoral response could be tremendously important for initial activation of myelin-specific T cells as well as for development of first clinical relapses, when the availability of CNS auto-antigen is still limited. Third and most importantly, our data implicate that independent of current B cell-oriented therapeutic approaches, inhibiting or modulating the auto-reactive humoral response or interfering with its downstream signaling may be of enormous, vastly unrecognized therapeutic potential for prevention of early relapses and disease progression in MS, NMO and related disorders.

## Electronic supplementary material

Below is the link to the electronic supplementary material.

Supplementary material 1 (PDF 570 kb)
