# Supplementary material for: Myelin-reactive antibodies initiate T cell-mediated CNS autoimmune disease by opsonization of endogenous antigen
Source: Acta Neuropathol. 2016 Mar 29;132:43–58. doi: 10.1007/s00401-016-1559-8 (PMC4911382; doi:10.1007/s00401-016-1559-8)
Supplement: Supplementary file 1 — Supplementary material 1 (PDF 570 kb) [file 401_2016_1559_MOESM1_ESM.pdf]

Supplemental material

Myelin-reactive antibodies initiate T cell-mediated CNS autoimmune disease by opsonization of endogenous antigen

Silke Kinzel[1*], Klaus Lehmann-Horn[2*], Sebastian Torke[1], Darius Häusler[1], Anne Winkler[1], Christine Stadelmann[1], Natalie Payne[3], Linda Feldmann[1], Albert Saiz[4], Markus Reindl[5], Patrice H. Lalive[6,7], Claude C. Bernard[3], Wolfgang Brück[1], Martin S. Weber[1,8]

[1]Department of Neuropathology, University Medical Center, Georg August University, Göttingen, Germany; [2]Department of Neurology, Klinikum rechts der Isar, Technische Universität München and Munich Cluster for Systems Neurology, Munich, Germany; [3]Monash Regenerative Medicine Institute, Multiple Sclerosis Research Group, Monash University, Melbourne, Australia; [4]Service of Neurology, Hospital Clinic, University of Barcelona, Barcelona, Spain; [5]Clinical Department of Neurology, Medical University of Innsbruck, Innsbruck, Austria; [6]Department of Clinical Neurosciences, Division of Neurology, University Hospital of Geneva, Geneva, Switzerland; [7]Department of Pathology and Immunology , Faculty of Medicine, University Hospital of Geneva, Geneva, Switzerland; [8]Department of Neurology, University Medical Center, Georg August University, Göttingen, Germany

* These authors contributed equally and are listed in alphabetical order

Address correspondence to: Martin S. Weber, MD
Department of Neuropathology
Department of Neurology
University Medical Center, Georg August University
Robert-Koch-Str. 40, 37099 Göttingen, Germany
Phone +49-551 39-7706
Fax +49-551-39-10800
E-mail: martin.weber@med.uni-goettingen.de

Supplemental table 1 Summary of clinical and histological results upon transfer of purified anti-MOG Ab 8.18C5 or isotype control Ab into WT mice

	animals with histological or clinical signs of EAE			
	number of animals with CNS inflammation	number of animals with clinical symptoms	mean max. EAE severity (range)	onset of EAE; mean days after 1st serum/ ab transfer (range)
WT – 8.18C5	0/5	0/5	n.a.	n.a.
WT – isotype control	0/5	0/5	n.a.	n.a.

Supplemental Figure 1

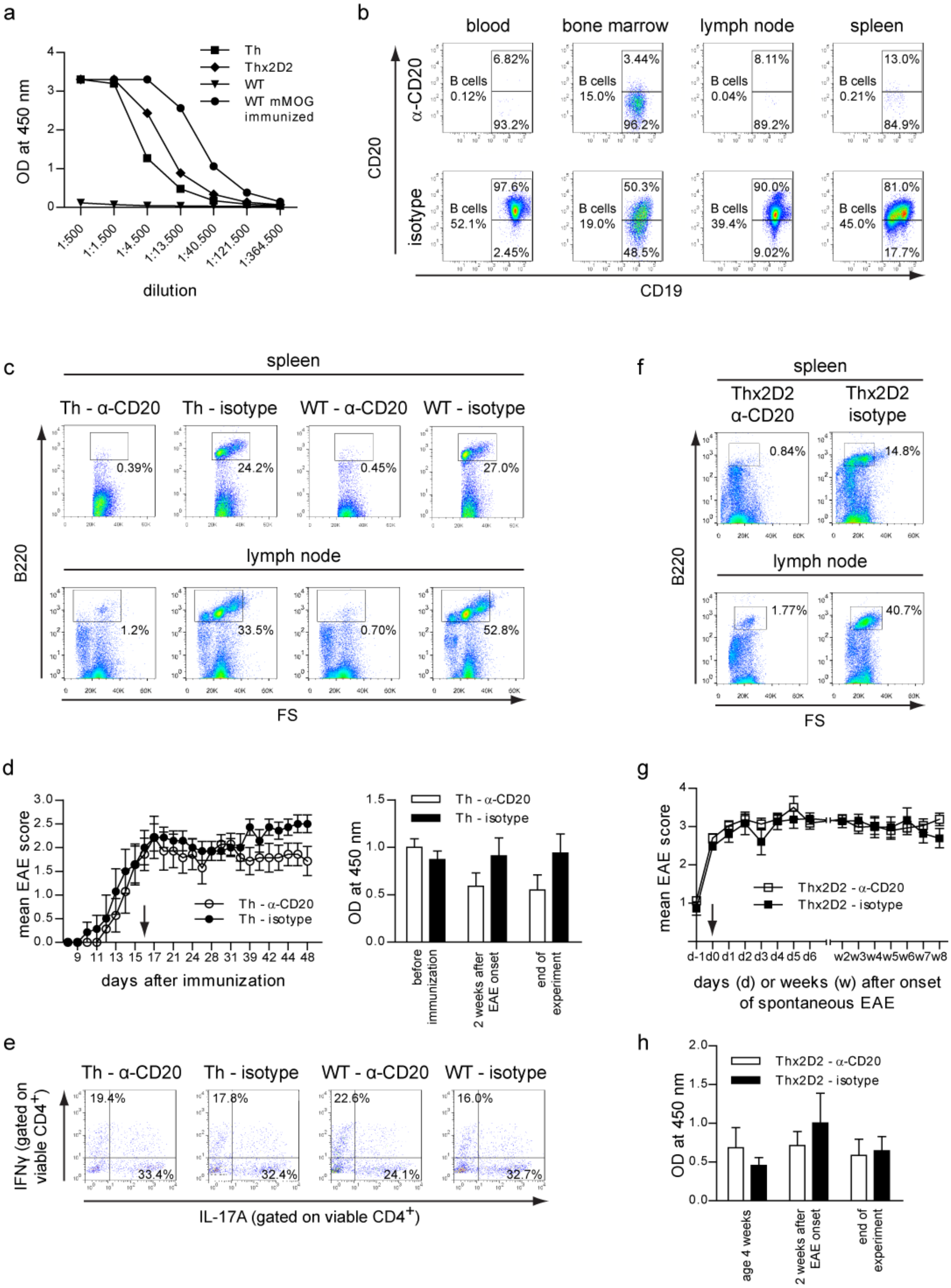

Suppl. Fig. 1 Depletion of B cells does not ameliorate established induced or spontaneous EAE when mMOG-specific Ab are present. **a** Detection of Ab against mMOG in Th, Thx2D2, WT and WT mice immunized with mMOG by ELISA at serial dilutions. **b** Frequency of CD19$^+$ B cells (left number) with corresponding CD20 expression in blood, bone marrow, lymph node and spleen of WT mice injected i.p. with anti (α)-CD20 or isotype control Ab once per week for 3 weeks. **c** Th or WT mice were injected with anti (α)-CD20 or isotype control Ab once per week, starting 3 weeks prior to immunization with mMOG. Frequency of B220$^+$ B cells in spleen (upper panel) and lymph node (lower panel) at the end of the experiment; $n = 10$ mice/group. **d** Th mice were immunized with mMOG and randomized to receive weekly anti-(α) CD20 or isotype control Ab at an EAE score $\geq$ 2. Initiation of anti-CD20 Ab treatment is depicted by arrow. Mean group EAE score ± SEM (left); p = ns (Mann-Whitney) and anti-mMOG IgG Ab serum levels determined by ELISA (dilution 1:40,500; right); $n = 7$ mice/group. **e** Th or WT mice were injected weekly with anti (α)-CD20 or isotype Ab starting 3 weeks prior to immunization with mMOG. Frequency of IFN-γ and IL-17A producing T cells isolated from the CNS at the end of the experiment; $n = 10$ mice/group. **f** Thx2D2 mice were injected weekly with anti (α)-CD20 or isotype control Ab starting 28 days after birth. Frequency of B220$^+$ B cells in spleen (upper panel) and lymph node (lower panel) at the end of the experiment. **g, h** Thx2D2 mice were randomized to receive weekly anti (α)-CD20 ($n = 23$) or isotype control Ab ($n = 22$) when spontaneous EAE was fully established (individual EAE score of $\geq$ 2). **g** Mean group EAE score ± SEM. Initiation of treatment is depicted by arrow; p = ns (Mann-Whitney). **h** Anti-mMOG IgG Ab serum levels determined by ELISA (dilution 1:13,500); p = ns (t test).

Supplemental Figure 2

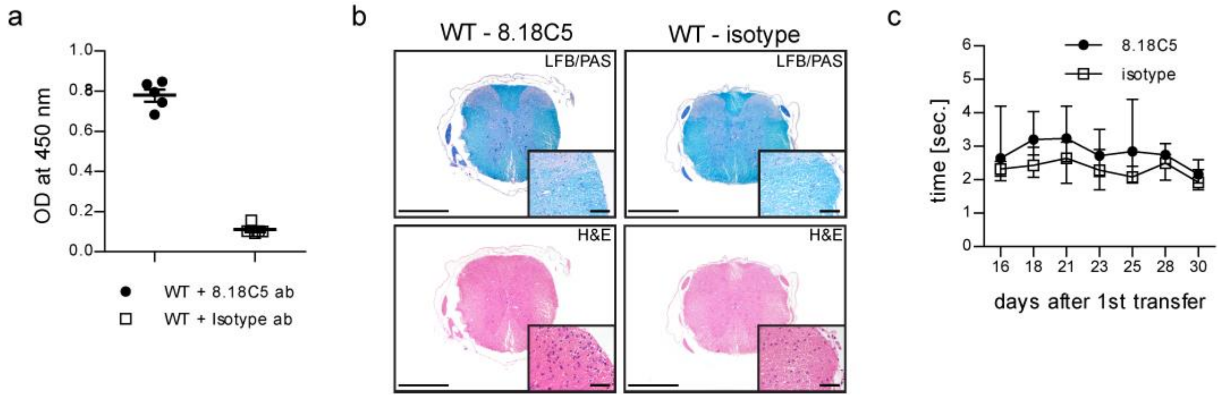

Suppl. Fig. 2 Transfer of anti-mMOG Ab containing serum or of purified anti-mMOG Ab triggers histologic EAE in 2D2 recipients, while WT recipients show no signs of CNS infiltration or demyelination. **a-c** Naïve WT mice were treated with 8.18C5 or isotype control Ab; n = 5 mice/group. **a** Anti-mMOG Ab serum levels determined by ELISA (dilution 1:13,500) at the end of the experiment. **b** Demyelination (LFB/PAS; upper panel) and overall inflammation (H&E; lower panel) of the spinal cord; one representative section of an 8.18C5 Ab-receiving vs. an isotype Ab-receiving animal is depicted. Scale bar overview = 500 µm, scale bar inlay = 50 µm. **c** Elevated beam test of WT mice receiving 8.18C5 or isotype control Ab. Indicated is the mean time in seconds (sec.) per group ± SEM required to traverse the beam; p = ns (Mann-Whitney).

Supplemental Figure 3

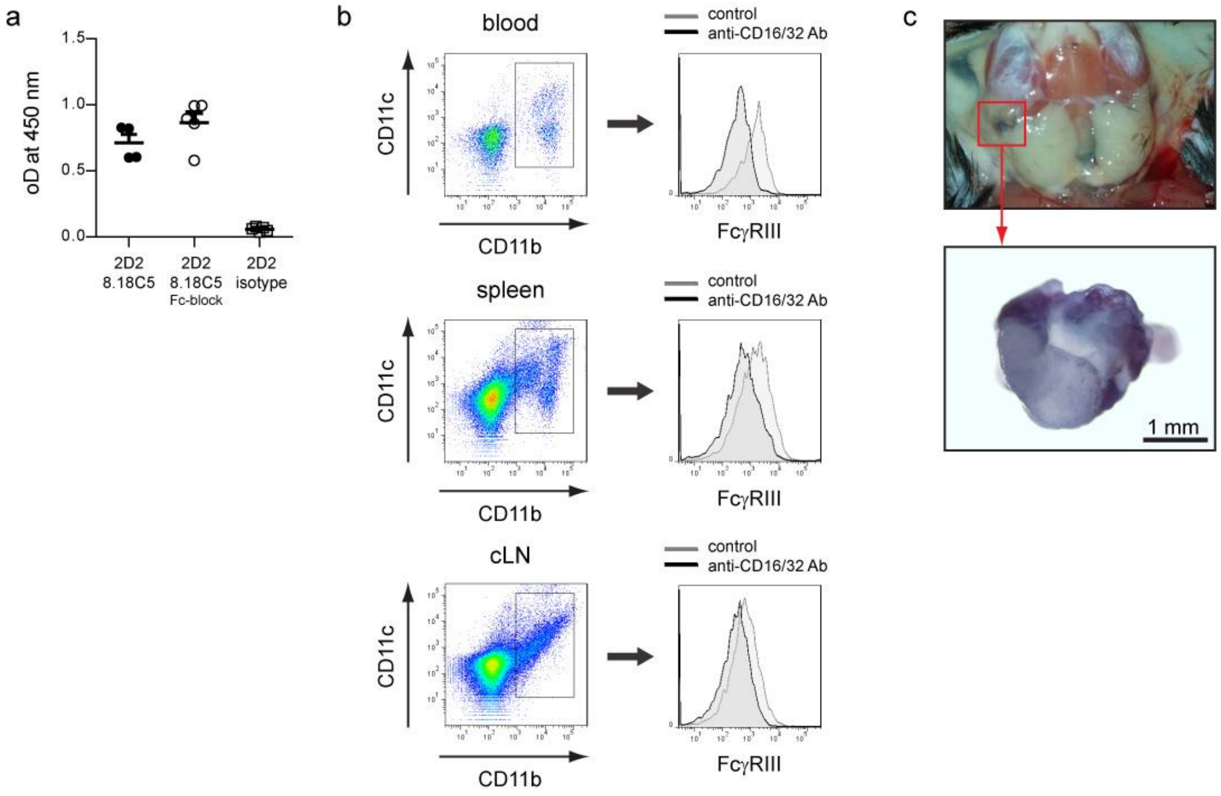

Suppl. Fig. 3 Drainage of intrathecally applied antigen and *in vivo* blockade of Fcγ receptor. **a** Naïve 2D2 mice were treated daily with Fc receptor blocking anti-CD16/CD32 or control Ab i.p. during the entire experiment. Starting at day (d)3, mice received three consecutive i.v. injections of 8.18C5 or control Ab. mMOG was injected intrathecally at d5. Anti-MOG Ab serum level was determined by ELISA (dilution 1:13,500) at the end of the experiment (d7). **b** Mice were treated three consecutive days with anti-CD16/CD32 Ab or control Ab. Representative FACS plots show the expression of Fcγ receptor III (right) on APC in blood, spleen and cervical lymph nodes (cLN). For analysis, cells were pre-gated on $CD11b^{+}/CD11c^{+}$ double positive cells (left); $n = 3$ mice/group. **c** Naïve WT mice were injected intrathecally with 10 µl of 10% Evans blue. Cervical lymph nodes were analyzed 1 hour after injection. The red square indicates the Evans blue-filled lymph node (upper picture); the lower picture shows the isolated lymph node in 40x magnification, scale bar =1 mm; $n = 3$.

Supplemental Figure 4

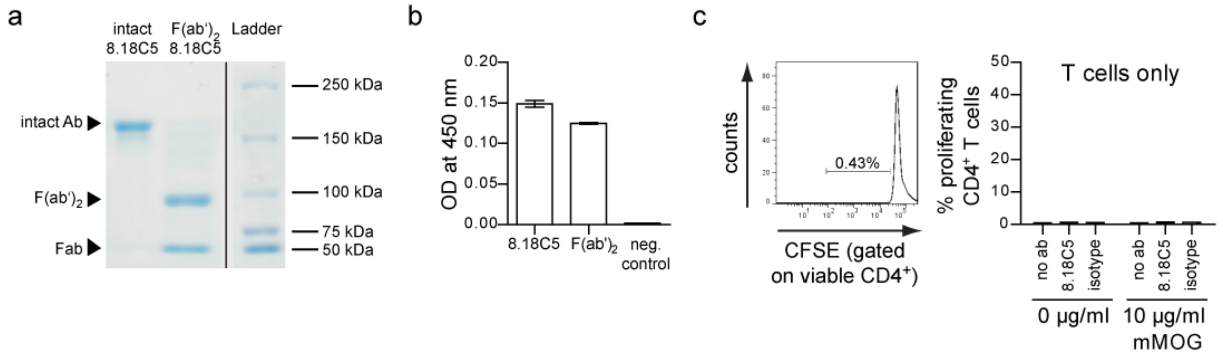

Suppl. Fig. 4 Functional analysis of 8.18C5 F(ab')$_2$ fragment and lack of a direct T cell stimulating effect of 8.18C5 Ab in the absence of APC. **a** 6% SDS-PAGE of intact 8.18C5 Ab (approx. 150 kDa) and its F(ab')$_2$ fragment (approx. 100 kDa) after digestion; an additional band at 50 kDa represents single Fab fragments. **b** mMOG ELISA of 8.18C5 F(ab')$_2$ fragments, intact 8.18C5 Ab and a negative control (unspecific Ab). Plate-bound Ab were detected with anti-mouse IgG Ab directed against the whole molecule. **c** CFSE-labeled MOG-specific 2D2 T cells cultured in the presence of mMOG and 8.18C5, or isotype control Ab. Proliferation of $CD4^+$ T cells determined by CFSE dilution (representative FACS plot, left; mean % of proliferating T cells in duplicates ± SEM, right).

Supplemental methods

Mice

MOG p35-55 TCR transgenic 2D2 mice were kindly provided by Dr. Kuchroo (Boston, USA). MOG Ig heavy chain knockin (Th) mice were kindly provided by Dr. Wekerle (Munich, Germany). WT C57BL/6 mice were purchased from Charles River (Sulzfeld, Germany). All murine experiments were carried out as approved by the government of Upper Bavaria (protocol number 55.2-1-54-2531-67-09) and the government of lower Saxony (protocol number 33.9-42502).

B cell depletion

B cell depletion was achieved by weekly i.p. injections of 200 µg of murine anti-CD20 or anti-ragweed isotype control Ab (both provided by Genentech, South San Francisco, USA) in 200 µl PBS.

Antigens and EAE induction regiments

Mouse MOG p35-55 (MEVGWYRSPFSRVVHLYRNGK) was synthesized by Auspep (Parkville, Australia). mMOG protein was kindly provided by C.C.A. Bernard and synthesized, purified and refolded as previously reported [4]. 8-10 week old female Th or WT mice were immunized subcutaneously with either a suboptimal dose of 35 µg/ml mMOG or an optimal dose of 50 µg/ml mMOG in CFA followed by 200 ng of pertussis toxin (Sigma-Aldrich, St. Louis, USA) i.p. at the day of immunization and 2 days thereafter. Alternatively, WT mice were immunized with 100 µg MOG p35-55 in CFA followed by two injections of 300 ng pertussis toxin. For spontaneous EAE experiments, Th mice were bred with 2D2 mice (Thx2D2). To induce EAE by transfer of MOG-specific Ab, a serum preperation containing anti-MOG Ab or mAb clone 8.18C5 were transferred into the tail vein of WT or 2D2 recipients. 150 µl Th serum

or control serum was injected twice a week up to a total of 5 injections; 150 µg 8.18C5 Ab or isotype control Ab (clone: MOPC-21, Bio X Cell, West Lebanon, USA) was injected twice a week up to a total of 10 injections. In recipients, no immunization or adjuvant treatment was added.

Evaluation of EAE

Mice were assessed for clinical signs of EAE as follows: 0 = no clinical disease, 1 = tail weakness, 2 = hind limb weakness, 3 = one paralyzed hind limb, 4 = two paralyzed hind limbs, 5 = moribund or dead. To evaluate balance and general motor function the elevated beam test was used. Therefore, mice were placed on a raised beam with a maximal height of 40 to 50 cm and a length of 100 cm. The time needed to traverse was measured. Mice were evaluated daily starting two weeks prior to the respective treatment.

Intrathecal injections

Mice were injected with 40 µg mMOG in 10 µl PBS or 10 µl of 10% Evans blue percutaneously into the cisterna magna with a 30-gauge needle in 45° anteflexion of the head.

Fc blocking in vivo

To block Fcγ receptors *in vivo*, mice were injected i.p. daily with 100 µg anti-CD16/CD32 Ab (Clone: 2.4G2, TONBO bioscience, San Diego, USA) in 100 µl 1xPBS starting two days prior to further treatments.

Generation of anti-MOG Ab containing serum

Serum containing high levels of pathogenic anti-MOG Ab or control serum was obtained from Th mice immunized with 100 µg mMOG (Th serum) or from WT mice immunized with 100 µg MOG p35-55 (control serum), respectively. 14 days after immunization, blood was obtained

by puncture of the left ventricle; serum was separated by centrifugation, pooled for further use and stored at -20°C.

Preparation and digestion of 8.18C5

Anti-MOG monoclonal Ab clone 8.18C5 was generated by hybridoma cells kindly provided by Dr. Christopher Linington. Hybridoma cells were cultured in complete medium (RPMI, 5-10% fetal calf serum, 50 U/ml penicillin, 50 µg/ml streptomycin, 2 mM L-glutamine, 1 mM sodium pyruvate, 0.05 mM β-mercaptoethanol) in large-scale flasks (Greiner bio-one, Kremsmuenster, Austria) and Ab was purified using rProtein A/Protein G Sepharose columns (rProtein A/Protein G GraviTrap, GE Healthcare, Little Chalfont, UK), according to manufacturer´s recommendations. 8.18C5 IgG was digested by ficin (Pierce Mouse IgG1 Fab and $F(ab')_2$ Preparation Kit, Thermo Scientific, Waltham, USA), according to manufacturer´s recommendations; integrity and binding capacity of 8.18C5 Ab and resulting 8.18C5 $F(ab')_2$ fragments was verified by 6% sodium dodecyl sulfate polyacrylamide gel electrophoresis (SDS-PAGE) under non-reducing conditions, followed by protein staining with Coomassie brilliant blue G250 (Biorad, Munich, Germany) and competitive anti-MOG enzyme linked immunosorbent assay (ELISA), respectively.

Assessment of T cell proliferation and differentiation in vitro

MACS-purified (Pan T cell Isolation Kit, Miltenyi, Bergisch Gladbach, Germany) CFSE-stained (CFSE Cell Division Tracker Kit, BioLegend, San Diego, USA) T cells from 2D2 mice were cultured with mMOG in the presence of 50 µg/ml 8.18C5 Ab, 8.18C5 $F(ab')_2$ fragment or isotype control Ab. 72 h thereafter, T cell proliferation and differentiation were evaluated by FACS.

Flow cytometric analysis

To obtain single cell suspensions of lymphoid tissues, respective spleens and lymph nodes were carefully dissected and passed through 70 µm strainer. Freshly obtained blood was mixed 1:2 with 1 mM EDTA and erythrocytes were lysed using BD Pharm Lyse Buffer, according to manufacturer's recommendation. B cells were stained for B220 (BioLegend) CD19 (BioLegend) and/or CD20 (BioLegend), T cells for CD3 and/or CD4 (all BD Bioscience). T cell differentiation was evaluated by intracellular cytokine staining for IFN-γ (eBioscience, San Diego, USA) and IL-17A (BD Bioscience) after 4-h incubation with PMA (50 ng/ml) and ionomycin (0.5 µg/ml) in the presence of monensin (1 µl Golgi-Stop per ml cell suspension, BD Bioscience). Dead cells were excluded using an Aqua Dead Cell Stain Kit (Invitrogen, Thermo Fisher Scientific, 405 nm excitation). CNS-infiltrating cells were isolated by discontinuous density gradient (Percoll) and stained in a similar manner [16] for CD3, CD4, IFN-γ and IL-17A. Myeloid APC were stained for CD11b and CD11c (both BioLegend). Expression of Fcγ receptor (FcγR) III was determined using anti-CD64 Ab (FcγRI, BioLegend).

ELISA for detection of MOG Ab

96-well plates were coated with 10 µg/ml conformational mMOG or conformational hMOG in 1xPBS overnight. Diluted samples were added for 2 h. After washing, plate-bound Ab of murine samples and 8.18C5 Ab were detected with HRP–conjugated anti-mouse IgG, directed against the Fc part of the bound Ab (1:6,000; Sigma-Aldrich) or against the whole molecule (1:5,000; Sigma-Aldrich).

Histology

Brain and spinal cord tissue was PBS-perfused and cryofixed or perfused and fixed with 4% paraformaldehyde and paraffin-embedded. To determine myelin loss and inflammatory infiltration, vertically- or horizontally-oriented sections were stained with Luxol Fast Blue and periodic acid Schiff (LFB/PAS) or hematoxylin and eosin (H&E), respectively.

Statistical analysis

EAE experiments were evaluated for significance using the Mann-Whitney test. The two-sided t test was used for all other statistical comparison. A value of $p < 0.05$ was considered significant. Data are presented as mean ± SEM.
